# Widespread genomic signatures of reproductive isolation and sex-specific selection in the Eastern Yellow Robin, *Eopsaltria australis*

**DOI:** 10.1093/g3journal/jkac145

**Published:** 2022-06-10

**Authors:** Lynna Kvistad, Stephanie Falk, Lana Austin

**Affiliations:** Biological Sciences, Monash University, Clayton, VIC 3800, Australia; Biological Sciences, Monash University, Clayton, VIC 3800, Australia; Deep Sequencing Facility, Max Planck Institute of Immunobiology and Epigenetics, Freiburg D-79108, Germany; Biological Sciences, Monash University, Clayton, VIC 3800, Australia

**Keywords:** speciation, hybrid zones, mitonuclear, Haldane’s Rule, sex-specific selection, sex chromosomes, inversions

## Abstract

How new species evolve is one of the most fundamental questions in biology. Population divergence, which may lead to speciation, may be occurring in the Eastern Yellow Robin, a common passerine that lives along the eastern coast of Australia. This species is composed of 2 parapatric lineages that have highly divergent mitochondrial DNA; however, similar levels of divergence have not been observed in the nuclear genome. Here we re-examine the nuclear genomes of these mitolineages to test potential mechanisms underlying the discordance between nuclear and mitochondrial divergence. We find that nuclear admixture occurs in a narrow hybrid zone, although the majority of markers across the genome show evidence of reproductive isolation between populations of opposing mitolineages. There is an 8 MB section of a previously identified putative neo-sex chromosome that is highly diverged between allopatric but not parapatric populations, which may be the result of a chromosomal inversion. The neo-sex chromosomal nature of this region, as well as the geographic patterns in which it exhibits divergence, suggest it is unlikely to be contributing to reproductive isolation through mitonuclear incompatibilities as reported in earlier studies. In addition, there are sex differences in the number of markers that are differentiated between populations of opposite mitolineages, with greater differentiation occurring in females, which are heterozygous, than males. These results suggest that, despite the absence of previously observed assortative mating, mitolineages of Eastern Yellow Robin experience at least some postzygotic isolation from each other, in a pattern consistent with Haldane’s Rule.

## Introduction

How species arise and persist is a central question in evolutionary biology. Much is unknown about the evolutionary and demographic processes by which populations diverge, including how geographic and spatial distributions, genomic architecture, and biochemical pathways lead to divergence, reproductive isolation (RI), and, ultimately, speciation (Seehausen *et al.* 2014; Harrison and Larson 2016; Ravinet *et al.* 2017; Wolf and Ellegren 2017). Classical speciation theory proposes that populations must necessarily diverge in allopatry such that geographically separated populations become genetically incompatible with each other, and that RI occurs upon secondary contact (Mayr 1942, 1963). Such genetic incompatibilities are elaborated in the Dobzhansky-Muller incompatibility models (DMIs), which states mutant alleles that have become fixed in isolation are not harmful to their native populations; these, however, become deleterious when introduced into new populations with incompatible genetic backgrounds (Dobzhansky 1936; Muller 1940, 1942). Additional theory and empirical examples have since suggested that populations may also diverge in parapatry or sympatry, although the frequency with which speciation with gene flow occurs is controversial, and it has since been proposed that divergence should rather be measured in terms of gene flow, recombination rate, migration, and selection rather than geography (Endler 1977; Nosil 2008; Feder *et al.* 2012; Via 2012; Cruickshank and Hahn 2014; reviewed in Harrison and Larson 2016; Wolf and Ellegren 2017).

Divergence may be driven through intrinsic factors, which occur within an organism and independently of the external environment (such as genomic incompatibilities), or extrinsic factors that are dependent on the external environment (such as local adaptation) (Abbott *et al.* 2013; Seehausen *et al.* 2014). In addition, divergence may also be driven by selection at different stages of the life cycle: prezygotic selection occurs before the formation of a zygote (such as behavioral or ecological factors that isolate populations and prevent them from interbreeding), while postzygotic selection acts after the formation of a zygote has occurred (such as reduced viability or fitness of a hybrid individual) (Wolf *et al.* 2010; Abbott *et al.* 2013; Seehausen *et al.* 2014). Identifying the factors contributing to speciation is complicated in that the number of diverged markers between populations increases after RI is achieved and gene flow between them has ceased (Nosil and Schluter 2011). Markers that diverge after RI has occurred have not contributed to speciation; thus, to be identified as drivers of speciation, loci must be shown to have diverged before populations experience RI (Nosil and Schluter 2011).

A key area of research in speciation genomics resides in understanding how many loci are implicated in the initial divergence between populations, how that number changes as divergence progresses, and how such loci are distributed across the genome. Varied strength of selection amongst loci means that those under strong selection can form discrete “islands of differentiation” against a background of less differentiated loci; such “islands” commonly form in genomic regions of low recombination (Noor and Bennett 2009; Feder *et al.* 2012; Nachman and Payseur 2012; Via 2012; Renaut *et al.* 2013; Cruickshank and Hahn 2014; Ortiz-Barrientos *et al.* 2016). However, such islands have also been predicted to be the result of heterogeneous linked selection and hitchhiking across the genome in areas of low recombination, rather than arising through direct selection on the differentiated loci (Burri 2017; Ellegren and Wolf 2017; Stankowski *et al.* 2019).

While genomic “islands” are commonly seen between diverged populations and may occur anywhere in the genome, it is unknown if the same loci or biochemical pathways tend to drive speciation across taxa. It is also unknown to what extent DMIs within the nuclear genome drive speciation, or rather if DMIs between the nuclear and cytoplasmic genomes are more prominent (Lima *et al.* 2019). Nevertheless, DMIs between mitochondrial DNA (mtDNA) and nuclear DNA (nDNA) have been suggested to be a common driver of speciation across a wide range of taxa (Hill 2016; 2017). The hypothesis that mtDNA is heavily involved in speciation is based on the facts that mtDNA does not undergo recombination and has an effective population size one-quarter of diploid nuclear loci; thus, mutations are theorized to accumulate in the mtDNA more readily than in nDNA (Gershoni *et al.* 2009; Burton and Barreto 2012). Highly deleterious variants are then predicted to be selected against through purifying selection, or otherwise compensated for through nuclear mutations (Rand *et al.* 2004; Stewart *et al.* 2008; Gershoni *et al.* 2009; Montooth *et al.* 2010; Burton and Barreto 2012). All mtDNA gene products interact with nuclear encoded gene products (via mitonuclear interactions) to produce the cellular metabolic pathway known as oxidative phosphorylation (OXPHOS); it is critical for an organism’s fitness the mtDNA and nDNA be coevolved with one another so that there are not deleterious effects to OXPHOS (Dowling *et al.* 2008; Burton and Barreto 2012). If 2 populations have separately evolved mtDNAs and nDNAs, and the populations later come into secondary contact with one another, there may be incompatibilities between these mtDNAs and nDNAs in a hybrid genomic background (Dowling *et al.* 2008; Burton and Barreto 2012; Barreto *et al.* 2018). Because of this, the interactions between the products of mtDNA and nDNA variation are predicted to lower fitness in the hybrid offspring of lineages that are in the process of diverging (Gershoni *et al.* 2009).

Because mtDNA is maternally inherited in most species, it undergoes evolution only in females; this may potentially lead to sex-specific fitness effects (Gemmell *et al.* 2004; Dowling *et al.* 2008; Innocenti *et al.* 2011; Camus *et al.* 2012; Beekman *et al.* 2014). Indeed, mitochondrial haplotypes have been found to incur sex-specific expression and fitness effects (Montooth *et al.* 2010; Innocenti *et al.* 2011; Mossman *et al.* 2016; Connallon *et al.* 2018). Such rapid accumulation of mutations in combination with uniparental inheritance may lead to mitochondrial haplotypes being locally adaptive (Dowling *et al.* 2008; Cheviron and Brumfield 2009; Boratyński *et al.* 2011, 2014; Toews *et al.* 2013).

In addition to mtDNA, sex chromosomes are also thought to play a strong role in divergence; this is true both for XY systems (such as mammals), where males are the heterogametic sex, and ZW systems (such as birds and butterflies), where females are heterogametic. Which is the heterogametic sex is not without evolutionary consequence, and results in different selective pressures on sexes, the chromosomal distribution of genes, and dosage compensation in XY vs ZW systems (Mank 2013; Dean *et al.* 2014; Rogell *et al.* 2014). This may be especially important for genes on sex chromosomes that may have epistatic interactions with mtDNA or otherwise signal mitonuclear status: there is a paucity of mitonuclear genes on the X chromosome compared to autosomes in mammals, but not the Z chromosome in birds (Drown *et al.* 2012; Hill and Johnson 2013; Dean *et al.* 2014; Hill 2014; Rogell *et al.* 2014).

Because it is hemizygous in females, the Z chromosome has greater exposure of recessive alleles in females compared to males, as well as a smaller effective population size, and the potential for faster evolution, lower recombination rate, and an accumulation of genes with sex-specific effects compared to autosomes (Orr 1993; Turelli and Orr 1995; Ellegren 2009; Qvarnström and Bailey 2009; Rheindt and Edwards 2011; Irwin 2018). When divergent populations hybridize, the hemizgametic sex is much more affected by recessive alleles that are located on a sex chromosome and incompatible with the rest of the genome than is the homogametic sex. This is because the homogametic sex has a second sex chromosome that may harbor a compatible allele, whereas no such second, potentially compatible allele exists in the hemizygous sex (Orr 1993; Turelli and Orr 1995). This pattern, where lower fitness is observed more frequently in the hemizygous than heterogametic sex in hybridizing lineages, is known as Haldane’s Rule (Haldane 1922). Genomic incompatibilities between diverging populations are thought to accumulate faster on the Z chromosome than elsewhere in the avian genome, and on sex chromosomes faster than autosomes more generally (Mank *et al.* 2007; Ellegren 2009; Qvarnström and Bailey 2009; Irwin 2018; Janoušek *et al.* 2019). A comparative analysis of passerines shows that during divergence, the first aspect of fitness loss in hybrids tends to be female sterility, followed by male sterility, female inviability, and finally male inviability, in a pattern consistent with Haldane’s Rule in ZW systems (Price and Bouvier 2002).

Biogeographic discordance between mtDNA and nDNA has been found in a variety of taxa, but there is debate over the mechanisms driving such patterns and how they might be dictated by the biology of the species in question (Toews and Brelsford 2012). Processes such as adaptive introgression and incomplete lineage sorting have been implicated in some systems, while demographic processes such as sex-biased dispersal have been used to explain patterns in others (Toews and Brelsford 2012). For instance, in birds, Haldane’s Rule predicts that hybrid females are unable to contribute to gene flow across avian hybrid zones as much as hybrid males do, because hybrid females are more likely to be sterile or inviable. In particular, female sterility prevents introgression of mtDNA more than it does to autosomes, and this pattern of differentiation of mtDNA but introgression of nDNA has been found in several natural avian systems (Tegelström and Gelter 1990; Helbig *et al.* 2001; Carling and Brumfield 2008; Rheindt and Edwards 2011; Hill and Johnson 2013; Gowen *et al.* 2014). However, the reduced introgression of mtDNA compared to autosomes has also been attributed to other mechanisms, such as sex-biased dispersal and local adaptation of mtDNA with genomic analyses being too insensitive to detect corresponding local adaptation of the nuclear genome (Petit and Excoffier 2009; Bonnet *et al.* 2017).

Understanding how mtDNA, sex chromosomes, and autosomes interact is a complex yet important aspect of understanding how populations diverge and new species arise. This study examines the genetic architecture and types of selection driving divergence in a bird that exhibits discordance between its mitochondrial and nuclear genomes in parapatric populations. The Eastern Yellow Robin, *Eopsaltria australis*, exhibits 2 mitochondrial lineages, inland and coastal, but the mechanisms driving divergence between these mitolineages remain largely unknown. To determine the genomic signatures of RI in the nuclear genome that may be driving speciation between mitolineages, genomic clines were calculated for loci across the nuclear genome. We assessed how many (if any) loci contribute to RI, how they are distributed throughout the genome, if sex chromosomes play a disproportionate role in divergence, and what types of selection may be driving RI. Furthermore, we compared the patterns of nuclear admixture in a hybrid zone, as well as patterns of nucleotide differentiation, divergence, and diversity between and within and between mitolineages and sexes so as to investigate any sex-specific signatures of selection. Finally, this article considers the role of mitonuclear interactions in driving speciation between mitolineages in the Eastern Yellow Robin.

## Materials and methods

### Study system

The Eastern Yellow Robin, *Eopsaltria australis* (EYR), is a small, common passerine that occurs along the eastern coast of Australia (Higgens and Peter 2002). This species has been found to be composed of 2 highly divergent (6.8% nucleotide substitution) mitolineages that occur parapatrically: the inland mitolineage occurs inland of the Great Dividing Range in Victoria and New South Wales, as well as throughout most of the species’ northern range in Queensland, while the coastal mitolineage occurs in coastal areas of Victoria and New South Wales (Pavlova *et al.* 2013). There is also a north-south gradient of nuclear population structure, perpendicular to the structure exhibited by mitochondrial divergence (Pavlova *et al.* 2013). The distributions of EYR mitolineages correlate with the environmental variables of maximum temperature of the warmest month and minimum precipitation of the driest month (Pavlova *et al.* 2013). Despite purifying selection acting across the mitochondrial genome, several codons exhibit evidence of positive selection, suggesting the range of each mitolineage may be influenced by local adaptation of mitochondrial haplotypes and thus sex-specific selection (Pavlova *et al.* 2013; Morales *et al.* 2015; Lamb *et al.* 2018).

Nuclear gene flow was previously found to occur between the 2 mitolineages through coalescent analyses and isolation-with-migration modeling in IMa2 using 8 autosomal microsatellites (Pavlova *et al.* 2013). Incomplete lineage sorting was ruled out as an alternative explanation for the discrepancy in population structure between mtDNA and nDNA because mtDNA haplotypes are distributed parapatrically for over 1,500 km (Pavlova *et al.* 2013). Vicariance was also considered unlikely as the Great Dividing Range is not thought to prevent gene flow (Pavlova *et al.* 2013). Coalescent analyses in IMa2 were later repeated with 400 nuclear loci; these found asymmetric gene flow from the coastal to inland mitolineages, as well as from south to north (Morales *et al.* 2017). However, isolation-with-migration models, including IMa2, have been shown through simulation modeling to chronically suggest asymmetric gene flow between populations that have none (Cruickshank and Hahn 2014). Morales *et al.* (2017) resolve the perpendicularity between the mitochondrial and nuclear population structures by suggesting that the species was separated into northern and southern allopatric populations during the late Pleistocene; it was during this time that mitochondrial and nuclear divergence occurred (Morales *et al.* 2017). Changes in climate later allowed for the introgression of the northern population southward through a path inland of the Great Dividing Range, while the southern population introgressed northward via its coastal side (Morales *et al.* 2017).

Little differentiation between mitolineages was initially found by Pavlova *et al.* (2013) using 8 autosomal microsatellites, though subsequent re-analyses by Morales *et al.* (2018) using over 60,000 SNPs showed there to be clusters of differentiation within the nuclear genome (Pavlova *et al.* 2013; Morales *et al.* 2018). These included a small but significant cluster of differentiated markers mapping to Zebra Finch (ZF) chromosome Z, and a much larger cluster mapping to ZF chromosome 1A (Morales *et al.* 2018). The larger cluster on ZF chromosome 1A harbors a disproportionately high number of genes involved in mitochondrial function when compared to the rest of the nuclear genome, and was found to occur in strong linkage disequilibrium (LD) with mitochondrial haplotype (Morales *et al.* 2018). However, clusters of mitonuclear genes outside of chromosome 1A were not tested for LD; neither were any nuclear–nuclear interactions, or genes unrelated to mitonuclear function. Omission of these analyses makes it difficult to determine if mitonuclear interactions between the mtDNA and chromosome 1A are stronger than other interactions, and thus be confirmed as drivers of speciation in EYR. Furthermore, the LD between the mtDNA and chromosome 1A was done by pooling all individuals of a mitolineage together; because no distinction was made for individuals differing by sex or geography, it is possible that factors outside of the hybrid zone (and thus unrelated to speciation) are responsible for the pattern.

**Fig. 1.**
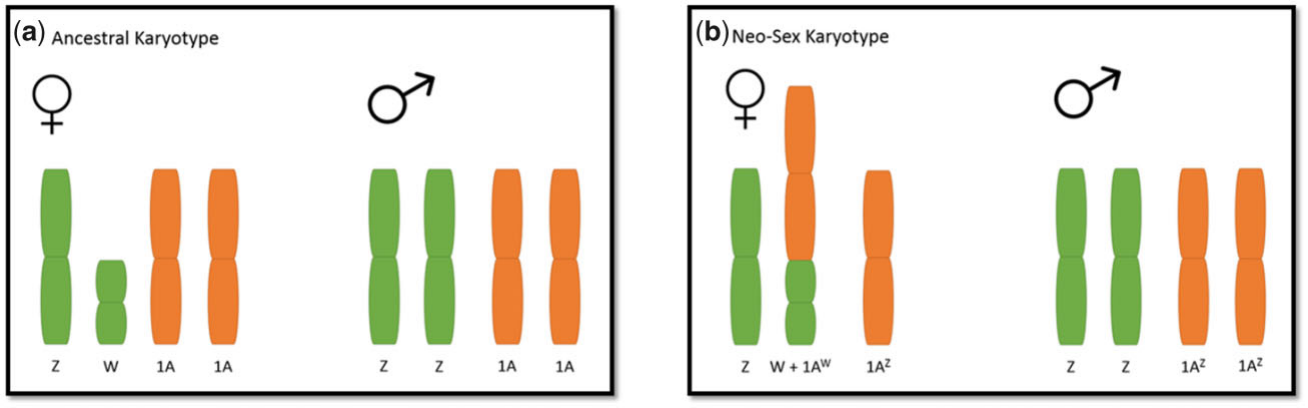
Eastern Yellow Robin neo-sex chromosome proposed model, a) depicts the ancestral karyotype where chromosome 1A occurs as an autosome independently of sex chromosomes W and Z, and b) depicts the current karyotype where one copy of chromosome 1A has fused with W, producing neo-sex chromosomes 1A^W^ and 1A^Z^ (in addition to sex chromosome Z).

The conclusions of mitonuclear interactions between mtDNA and chromosome 1A driving speciation were put further into doubt when partial assembly of a reference genome of an individual EYR of the inland mitolineage found that what is homologous autosome 1A in ZF may, in fact, be a putative neo-sex chromosome in EYR. Scaffolds of the EYR inland genome were considered to be W-linked if they were absent in males and had half of the read depth in females relative to the average read depth of scaffolds; scaffolds were considered as Z-linked if they had half of the read depth in females and a normal read depth in males (Gan *et al.* 2019). Scaffolds were then mapped to the ZF reference genome; 1,138 W-linked scaffolds and 179 Z-linked scaffolds were mapped to ZF chromosome 1A (Gan *et al.* 2019). Thus, it was hypothesized that what is chromosome 1A in ZF is a neo-sex chromosome in EYR. Fusion of one copy of ancestral autosome 1A with ancestral W is the proposed mechanism for this pattern; the copy of 1A that is fused with W would become 1A^W^, and the copy of 1A that remains would, by default, become 1A^Z^ (Fig. 1) (Gan *et al.* 2019). Because W and 1A^W^ are maternally inherited along with mtDNA, all W-linked markers must, by definition, be in complete LD with mtDNA. Because the presumed neo-sex chromosome nature of chromosome 1A was not taken into account when calculating LD between mtDNA and chromosome 1A in Morales *et al.* (2018), it is unclear how much LD will remain between mtDNA and 1A^Z^ when 1A^W^ is removed from the data (Morales *et al.* 2018).

### Field sampling

**Fig. 2.**
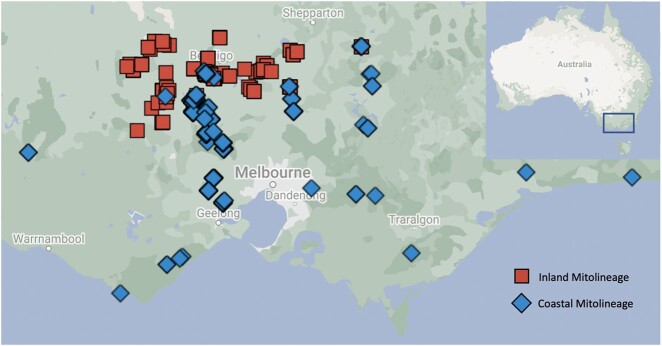
Map of sampled birds in Victoria. Red squares indicate birds of the inland mitolineage, and blue diamonds indicate birds of the coastal mitolineag.

A total of 442 individuals were sampled in Victoria, Australia (Fig. 2 and Supplementary Appendix 1). Sites were defined by the name of the forest, park, or reserve where sampling occurred. To estimate the distribution of inland and coastal mitolineages, widespread sampling took place throughout Victoria. To produce a detailed distribution of each mitolineage where they co-occur in a narrow hybrid zone of approximately 20–40 km wide, intensive local sampling was conducted in central Victoria; however, perfectly continuous sampling was prevented by anthropogenic landscape fragmentation. Individuals were defined as inland if they had an inland mtDNA haplotype, and as coastal if they had a coastal mtDNA haplotype. Sites were defined as “pure” where only one mitolineage occurred (i.e. allopatric sites), and “hybrid” at sites where both mitolineages occurred (i.e. sympatric sites). This created 4 categories of populations: pure inland, hybrid inland, hybrid coastal, and pure coastal (Table 1). Hybrid sites were restricted to those in which individuals of both mitolineages occurred within 5 km of one another; 5 km is within the known dispersal distance of EYRs, and so should not overestimate the size of the hybrid zone (Debus and Ford 2012, Higgens and Peter 2002). Sites where only one mitolineage occurred were classified as “pure” regardless of their proximity to the opposite mitolineage; even if birds of the opposite mitolineage occurred within dispersal distance of a pure site, the fact that none were sampled there strongly suggests that the distribution of mitolineages is nonrandom.

**Table 1.** Sample sizes for Victorian populations of Eastern Yellow Robin.

Sex	Pure inland	Hybrid inland	Hybrid coastal	Pure coastal
Males	72	54	55	110
Females	41	24	40	46

Pure inland refers to individuals of the inland mitolineage that were sampled at sites where only the inland mitolineage occurs; hybrid inland refers to individuals of the inland mitolineage that were sampled at sites where both inland and coastal mitolineages occur. Likewise, pure coastal refers to individuals of the coastal mitolineage that were sampled at sites where only the coastal mitolineage occurs; hybrid coastal refers to individuals of the coastal mitolineage that were sampled at sites where inland and coastal mitolineages both occur.

Individuals were captured in mist-nets and traps, issued with a leg band in compliance with the Australian Bird and Bat Banding Scheme (ABBBS), and banded with an additional colored leg band for future identification (ABBBS authority number 2870). Individuals were sampled and processed in accordance with Monash University approved ethics BSci 2021-20, 2007-07, and 2015-20. Individuals sampled and processed on land owned by Parks Victoria and the Department of Environment, Land, Water, and Planning were done in compliance with Wildlife Permit numbers 10007165, 10005919, 10005514, and 10007910. Call play-back was used to attract birds to the nets and traps; this can result in sex-biased sampling as males may be more likely than females to respond to call play-back. Individuals were blood sampled by taking up to 50 μl of blood from the brachial vein. Sampling was done on adult birds between autumn 2016 and spring 2018; these were added to samples included in previously published work to form the dataset for this study (Pavlova *et al.* 2013; Morales *et al.* 2017, 2018).

### Genomic mapping and synteny

DNA was extracted from blood for genomic analyses using the DNeasy Blood and Tissue extraction kit and following the manufacturer’s instructions (Qiagen, Germany). The sex and mitolineage of each individual was determined using sexing PCR and ND2 sequencing, respectively (following Pavlova *et al.* 2013). Genome-wide short-read (approximately 69 bp) SNP sequences were produced by Diversity Arrays Technology (DArTseq); these data are available on FigShare (Jaccoud *et al.* 2001; Kilian *et al.* 2012; Georges *et al.* 2018). Short-read sequences were mapped to the male Zebra Finch (ZF) reference genome *Taeniopygia guttata* 3.2.4 in BLAST 2.7.1 (Altschul *et al.* 1990; Camacho *et al.* 2009; Warren *et al.* 2010). Default parameters for short-read sequences were used (Blastn-short, word length of 11, gap open of 5, gap extend of 2, penalty of 3, and reward of 2), as well as an E-value of 1 × 10^−4^ to identify high quality hits (i.e. a hit can be expected to occur by chance no more than 1 × 10^−4^ times). Loci that did not adhere to these criteria were filtered out, as were loci that mapped to more than one location. Only biallelic markers were included. The male ZF reference genome consists of 32 autosomes plus the Z chromosome and contains approximately 1.2 GB of sequences; EYR DArT tags mapped to all chromosomes on the ZF reference genome and linkage groups LGE2 and LGE22, but not linkage group LGE5 (Warren *et al.* 2010). The number of mapped DArT tags per chromosome was proportional to the size of the chromosome (Supplementary Appendix 2).

Because markers were mapped to a homogametic male ZF reference genome, markers were unable to be mapped to the W chromosome. While intrachromosomal arrangements are common in birds, interchromosomal rearrangements are thought to be rare and synteny is considered high; for this reason, a high degree of synteny was assumed to occur between ZF and EYR (Ellegren 2010; Zhang *et al.* 2014; Singhal *et al.* 2015; Kawakami *et al.* 2017). Supporting this assumption is previous work that has found that EYR DArT tags mapped to similar positions to both ZF and Collard Flycatcher (CF) for most chromosomes, including chromosome 1A (Warren *et al.* 2010; Ellegren *et al.* 2012; Morales *et al.* 2018). Synteny is also known to be very high between ZF, CF, and chicken, which further suggests that it is reasonable to assume that synteny would also be very high between ZF and EYR (Backström *et al.* 2008; Stapley *et al.* 2008; Kawakami *et al.* 2014). Mapping to the ZF genome allows for comparison to previous work on the EYR such as in Morales *et al.* (2018), which chose the ZF genome as a reference instead of CF due to its more advanced annotation (Morales *et al.* 2018). To account for possible mis-mapping of markers onto the W chromosome, confirm synteny between ZF and EYR, and provide quality control for mapping errors despite assumed synteny, DArT tags were also mapped to W- and Z-behaving scaffolds of the inland mitolineage EYR draft genome. To help control for the partial assembly of the inland EYR genome and filter for W-behaving markers that did not map to a W-behaving scaffold, individual markers were further classified as W-behaving using population genetic behavior. Any markers that either mapped to W-behaving scaffolds in the EYR inland draft genome or else had W-behavior were removed from the dataset.

### Population-specific marker behavior

Because the markers that map to ZF autosome 1A are thought to occur on a neo-sex chromosome in EYR, markers that map to ZF chromosome 1A were categorized according to population genetic marker behavior. Markers were categorized as 1A^W^ if they occurred in females but were missing data in males, and as 1A^Z^ if they occurred as homozygous in females and homozygous or heterozygous in males. To prevent categorizing monomorphic, potentially autosomally behaving markers as 1A^Z^, markers were required to exhibit heterozygosity in a minimum of 10% of males. Error filters, such as those of missing data and homozygosity in females, were set at 10%; a cutoff of 10% was chosen as a balance between classification stringency while retaining sufficient data despite genotyping error (such as missing data). Marker categorization was done separately for each population (i.e. pure inland, hybrid inland, hybrid coastal, and pure coastal) in case marker behavior differed between populations. All markers that were classified as W-behaving in all populations mapped to W-behaving scaffolds in the EYR inland draft genome; likewise, all markers that were classified as Z-behaving in all populations mapped to Z-behaving scaffolds. Markers that were not able to be cleanly categorized as either 1A^W^ or 1A^Z^ due not meeting filter criteria in all populations were not included in this study.

### Marker differentiation and divergence

To help identify markers that may be acting as barriers to gene flow between EYR lineages, *F*_ST_ and *D*_XY_ were calculated between hybrid inland and hybrid coastal populations on all markers mapping to ZF autosomes (excluding 1A) and chromosome Z, as well as markers mapping to ZF chromosome 1A and categorized as 1A^W^ or 1A^Z^; *π* was calculated for these markers within populations. *F*_ST_ is a statistic commonly used to measure allelic frequency differentiation between 2 populations, and was calculated per SNP (Wright 1931, 1943). However, low levels of within-population polymorphism can inflate *F*_ST_ when there is no true differentiation between populations, and so *D*_XY_ and *π* were calculated as additional measures of population divergence and diversity (Charlesworth 1998; Cruickshank and Hahn 2014). *D*_XY_ measures the average number of nucleotide differences in sequences between 2 populations, while *π* measures within-population nucleotide diversity in a sequence; *D*_XY_ and *π* were calculated using DArT tag sequences using an overlapping sliding window approach in 100 kB windows with an overlap of 50 kB using a custom script in R (Nei and Li 1979; R Core Team 2017; Morales *et al.* 2018). Due to the low coverage and thus noncontinuous nature of DArT tags across the ZF genome, a relatively large window of 100 kB was needed in order to include multiple sites; overlapping sites helps insure against spurious results that may occur due to lower coverage in some windows than others. To help identify loci that are diverged between mitolineages but are not necessarily barriers to gene flow, such as those that may be involved in other local adaptation or drift outside of the hybrid zone, these statistics were also calculated between pure inland and pure coastal populations. To account for sex difference in markers that may be contributing to divergence between mitolineages, *F*_ST_, *D*_XY_, and *π* statistics were run separately for each sex. To test if any sex-specific patterns found between mitolineages in females were due to sampling error from smaller sample sizes, iterations of randomly subsampled males (creating sample sizes equal to those in females) were conducted. All markers were run as diploid. Markers mapping to ZF autosomes (excluding 1A) and chromosome Z were filtered to exclude those with ≥50% missing data per pairwise comparison; markers mapping to ZF chromosome 1A had additional filters allowing only for markers with ≤10% missing data to ensure correct population genetic behavior assignment. Because *F*_ST_ depends on the level of polymorphism within a population, and because sampling error may inflate values for markers at which polymorphism occurs at low frequency, markers with a minor allele frequency (MAF) of ≤0.05 were excluded from *F*_ST_ analyses. MAF was not included as a filter for either *D*_XY_ or *π* because these statistics are independent of level of polymorphism. Because markers classified as 1A^Z^ allowed for a 10% error rate, heterozygous genotypes occurred at low rates in these markers in females due to genotyping error, even though females are expected to be homogametic; in order to maximize data, these markers were also run as diploid.

To confirm the presence of any markers that may be under sex-specific selection, *F*_ST_ was also calculated between sexes within populations. To further test if greater differentiation between mitolineages in one sex than the other was due to sampling error or other spurious results, the distribution of observed *F*_ST_ values were compared to those predicted via permutated populations of equal size (following Ruzicka *et al.* 2020). Correlation between *F*_ST_ and % of missing data per marker was tested to examine if high *F*_ST_ values were inflated by missing data. A threshold of *F*_ST_ ≥ 0.2 was chosen to define markers exhibiting strong differentiation because 0.2 was above the 99th percentile of differentiation in all population comparisons, and because delimitating a specific *F*_ST_ value rather than a percentile allowed for comparison between several population iterations where the number of differentiated markers varied. In addition, although all distributions will have markers above a particular percentile, a given distribution of *F*_ST_ does not necessitate that any values be above a particular number. While degree of differentiation can also be determined through *F*_ST_ outlier tests, these are problematic for speciation studies as current outlier detection methods were developed to identify single loci involved in local adaptation, and are not appropriate for systems with population structure, such as hybrid zones, because of the high risk of identifying false positives (Bierne *et al.* 2011; Narum and Hess 2011; Bierne *et al.* 2013; de Villemereuil *et al.* 2014).

### Barrier loci and population admixture

Genomic clines are a method of measuring if individual loci contribute toward RI and introgression (Gompert and Buerkle 2009). This is achieved by calculating observed genotype frequency in each parent population, and from this, estimating the frequencies of genotypes in hybrid populations under a neutral expectation of unrestricted gene flow through permutation of genotypes for each individual at each locus (Gompert and Buerkle 2009). Observed hybrid zone genotype frequencies that are outside of those predicted under neutral expectation are presumed to be so due to reduced or absent gene flow between parent populations, such as would be caused by RI (Gompert and Buerkle 2009). To test for RI between EYR lineages and examine the genetic architecture involved in any such RI, genomic clines were produced using maximum likelihood modeling in the R package *Introgress* (Gompert and Buerkle 2009, 2010; R Core Team 2017). The permutation model was used, with 1,000 replications per locus category. All iterations for each locus category were completed separately for males and females.

Genomic clines were calculated for a subset of markers sampled from across the genome. Markers were filtered to retain those that have ≤10% missing data to minimize imputation and thus spurious results. To account for potential linkage and avoid overestimating the number of markers under selection, 1 marker per 100 kB was subset from markers mapped to ZF autosomes (excluding 1A) and Z. Markers on 1A^Z^ were not filtered for linkage due to the relatively small number of SNPs and inherent uncertainty in the genomic architecture of that chromosome. Although it is advisable to include markers with fixed differences between parent populations insofar as is possible so that the model does not have to take uncertainty into consideration, it was not sensible to include this filter because there were only 3 markers with fixed differences between pure inland and pure coastal females, and none for males (Gompert *et al.* 2012). There were differences in the identities of markers with high degrees of differentiation between mitolineages in hybrid populations compared to those in pure populations, even if allelic frequencies were not completely fixed; higher rates of allelic fixation between mitolineages in hybrid than parental (or pure) populations may be due to differences in selection regimes and polymorphism in different populations. Choosing markers based on their behavior in parental populations will thus not necessarily inform on the hybrid zone dynamics of RI in this system (Supplementary Appendix 3). Because previous work suggested nuclear gene flow could occur between mitolineages through male but not female EYRs, genomic clines were run separately per sex to test whether females had different loci and selection regimes contributing to speciation compared to males (Pavlova *et al.* 2013). Markers that map to ZF autosomes (excluding 1A) were run as codominant. Markers that map to ZF Z, as well as Z-behaving markers that map to 1A, were treated as haploid in females and codominant in males.

Finally, to test the degree of admixture between inland and coastal nuclear alleles within the hybrid zone, *Introgress* was also used to calculate how markers of differing ancestries were admixed at each locus per individual. Such ancestry analyses indicate if an individual has inherited 2 inland alleles, 2 coastal alleles, or one of each at a particular locus. As with the clines, ancestry analyses were run separately on each sex.

## Results

### Population-specific marker behavior

A total of 172,268 DArT tags (the sum of all markers across all individuals) were mapped to the ZF genome. Of those, 89,002 were unique hits that mapped to only one genomic location, and 76,894 of those mapped to known chromosomes. Of the 66,998 markers that mapped to autosomes (excluding chromosome 1A) in ZF, 23 mapped to W-behaving scaffolds while an additional 87 had W-linked population genetic behavior; 152 markers mapped to Z-behaving scaffolds in EYR. Additionally, 20 markers that mapped to the Z chromosome in ZF mapped to the W-behaving scaffolds in EYR. Of the 4,615 markers that mapped to the Z chromosome in ZF, 10 mapped to W-behaving scaffolds in EYR and 3,495 mapped to Z-behaving scaffolds. A total of 5,280 markers mapped to ZF autosome 1A, with 336 of those classified as 1A^W^ and 302 as 1A^Z^ based on population genetic behavior. The rest of the markers that mapped to ZF autosome 1A did not have clear 1A^W^ or 1A^Z^ behavior, and so were not included in analyses here. All markers classified as 1A^W^ mapped to W- behaving scaffolds in the EYR draft genome, and all markers classified as 1A^Z^ mapped to Z- behaving scaffolds. Patterns of missing data tended to vary by population or sex (i.e. markers with high amounts of missing data in one group of individuals may have low amounts of missing data in another) (Supplementary Appendix 3).

### Divergence throughout the genome but particularly high on 1A^Z^

**Fig. 3.**
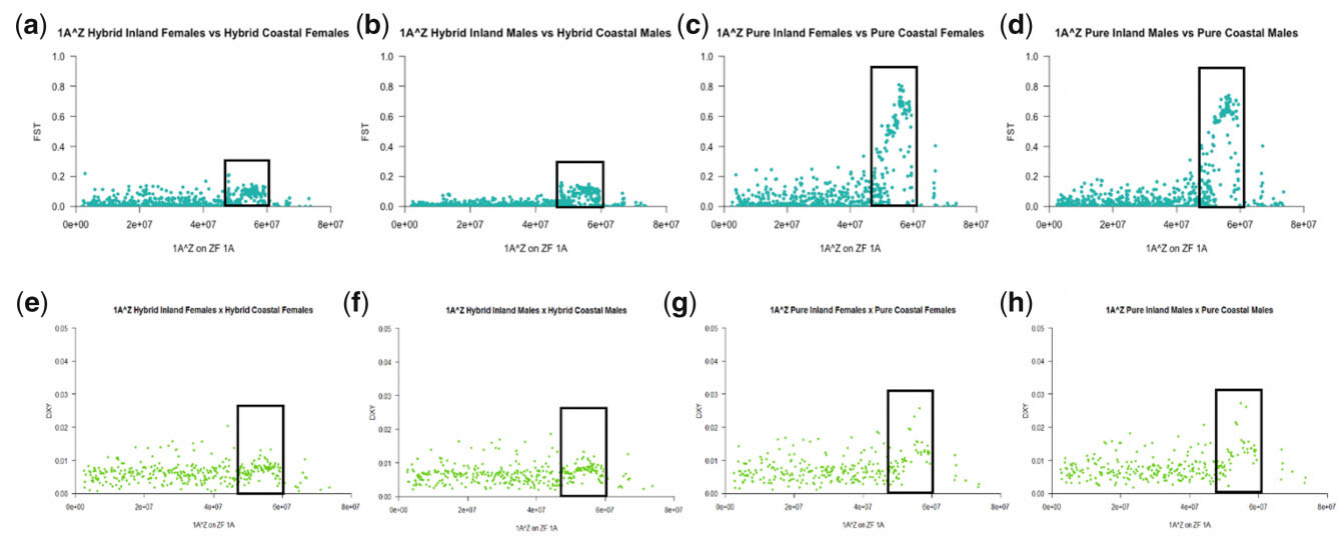
Differentiation and divergence between mitolineages for 1A^Z^ markers. Plots show *F*_ST_ values for a) females in the hybrid zone, b) males in the hybrid zone, c) females in pure populations, and d) males in pure populations; and *D*_XY_ values for e) females in the hybrid zone, f) males in the hybrid zone, g) females in pure populations; and h) males in pure populations. The x-axis shows marker position as mapped to Zebra Finch chromosome 1A. The markers in the black outlined box represent genomic region 1A^Z^* (51–59 MB).

1A^W^ markers had very high *F*_ST_ and *D*_XY_ values between mitolineages, both for hybrid and pure populations, indicating pronounced divergence (Supplementary Fig. 1). They also showed very low nucleotide diversity in each population (Supplementary Fig. 2, a–d). 1A^Z^ markers showed similar results in males and females, with low nucleotide diversity in all populations, and particularly low *π* in pure coastal individuals between marker positions 51–59 MB (Supplementary Fig. 2, e–l). There was little overall differentiation in 1A^Z^ markers between hybrid inland and hybrid coastal populations, although there was minor differentiation as well as an absence of markers with low *D*_XY_ values on 1A^Z^ between 51 and 59 MB (Fig. 3, a, b, e, and f). However, the differentiation in this same region was extreme between pure inland and pure coastal populations; again, there was a lack of markers here with low *D*_XY_ values, as well as a small increase in values relative to the other *D*_XY_ values along the chromosome (Fig. 3, c, d, g, and h). The region of 51–59 MB on 1A^Z^ will henceforth be referred to as 1A^Z^*.

**Fig. 4.**
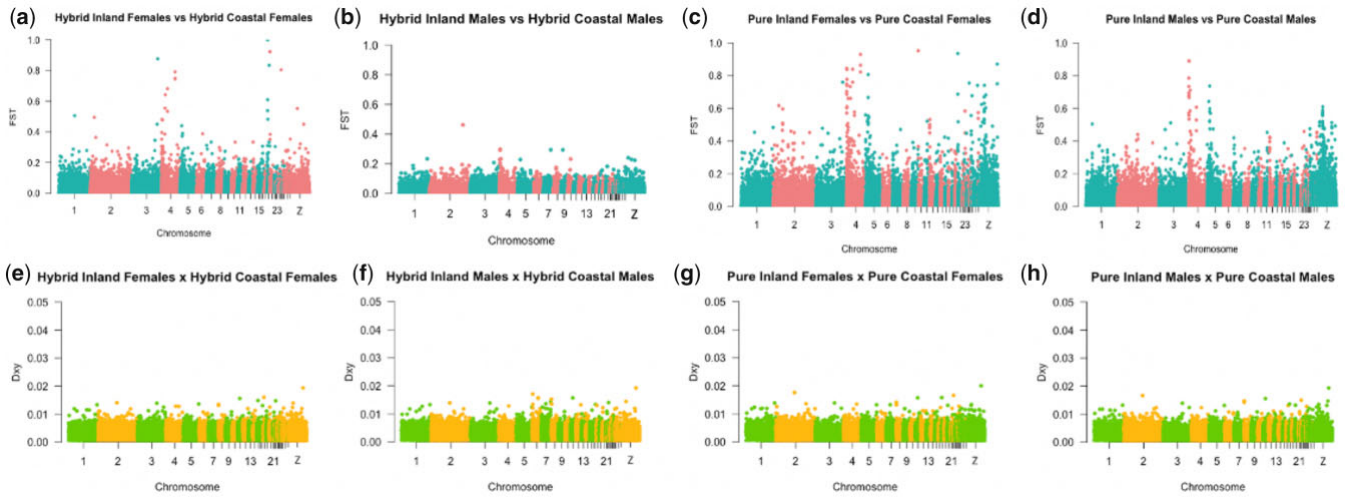
Differentiation and divergence between mitolineages for markers mapping to ZF autosomes (excluding 1A) and chromosome Z. Plots show *F*_ST_ values between a) females and b) males in the hybrid zone; *D*_XY_ values between c) females and d) males in the hybrid zone; *F*_ST_ values between e) females and f) males in pure populations; and *D*_XY_ values between g) females and h) males in pure populations.

For the rest of the genome, approximately 15 times as many markers exhibited greater differentiation between hybrid inland and hybrid coastal females than those populations of males; sex discrepancies in differentiation between pure inland and pure coastal birds was attributed to sampling error (Table 2, Fig. 4, and Supplementary Fig. 3). There was greater differentiation between pure inland and pure coastal populations than between hybrid inland and hybrid coastal populations, particularly on chromosomes 4, 5, and Z (Table 2 and Fig. 4). Patterns of nuclear diversity were similar for both sexes and were maintained across the genome (Supplementary Fig. 4). The observed distribution of *F*_ST_ values between mitolineages in hybrid populations was higher than expected when compared to permuted populations; this was true for iterations of each sex, indicating that the observed differentiation is not spurious (Supplementary Fig. 5, c and d). The distributions of *F*_ST_ values between hybrid inland females and hybrid coastal females and that between hybrid inland males and hybrid coastal males were confirmed to be different through a Mann–Whitney test (*P* < 0.001).

**Table 2.** Population summary of differentiated markers.

Populations	Marker type	No. of loci *F*_st_ >= 0.2
Hybrid inland females vs hybrid coastal females	Autosomal	182
Hybrid inland males vs hybrid coastal males	Autosomal	12
Overlap hybrid inland females vs hybrid coastal females and hybrid inland males vs hybrid coastal males	Autosomal	5
Hybrid inland females vs hybrid coastal females	Z	13
Hybrid inland males vs hybrid coastal males	Z	0
Overlap hybrid inland females vs hybrid coastal females and hybrid inland males vs hybrid coastal males	Z	0
Hybrid inland females vs hybrid coastal females	1A^Z^	3
Hybrid inland males vs hybrid coastal males	1A^Z^	0
Overlap hybrid inland females vs hybrid coastal females and hybrid inland males vs hybrid coastal males	1A^Z^	0
Hybrid inland females vs hybrid inland males	Autosomal	32
Hybrid coastal females vs hybrid coastal males	Autosomal	19
Overlap hybrid inland females vs hybrid inland males and hybrid coastal females vs hybrid coastal males	Autosomal	10
Hybrid inland females vs hybrid inland males	Z	7
Hybrid coastal females vs hybrid coastal males	Z	5
Overlap hybrid inland females vs hybrid inland males and hybrid coastal females vs hybrid coastal males	Z	3
Pure inland females vs pure coastal females	Autosomal	581
Pure inland males vs pure coastal males	Autosomal	384
Overlap pure inland females vs pure coastal females and pure inland males vs pure coastal males	Autosomal	384
Pure inland females vs pure coastal females	Z	123
Pure inland males vs pure coastal males	Z	149
Overlap pure inland females vs pure coastal females and pure inland males vs pure coastal males	Z	72
Pure inland females vs pure coastal females	1A^Z^	121
Pure inland males vs pure coastal males	1A^Z^	109
Overlap pure inland females vs pure coastal females and pure inland males vs pure coastal males	1A^Z^	104

Overlap between populations for the number of markers that are differentiated with at least F_ST_ ≥0.2. The total number of markers was 66,998 for autosomes, 4,615 for the Z chromosome, and 302 for putative neo-sex chromosome 1A^Z^.

Although greater differentiation was observed between mitolineages for females than males in the hybrid zone, the identity of the markers that were differentiated between mitolineages in males did not often overlap with the identity of those in females. This may be attributable to different selection regimes acting on females than males, or to sampling error (Table 2). Scattered differentiation of markers was also observed between sexes within the same population; greater differentiation was observed between hybrid inland females and hybrid inland males than between hybrid coastal females and hybrid coastal males. There was a slight concentration of markers on chromosome 19 exhibiting differentiation between sexes in the hybrid inland population (Supplementary Fig. 6a). However, the observed distribution of *F*_ST_ values between sexes did not differ from those of permuted populations for either hybrid inland or hybrid coastal populations, suggesting the observed differentiation is too small to skew the overall distribution, or may otherwise be due to sampling error (Supplementary Fig. 5,a and b). While *F*_ST_ did tend to increase with proportion of missing data for lower values of differentiation, markers that were highly differentiated were not skewed toward having missing data. This was true between mitolineages for both sexes, as well as between sexes within a population (Supplementary Fig. 7).

### Signatures of reproductive isolation across the genome

**Fig. 5.**
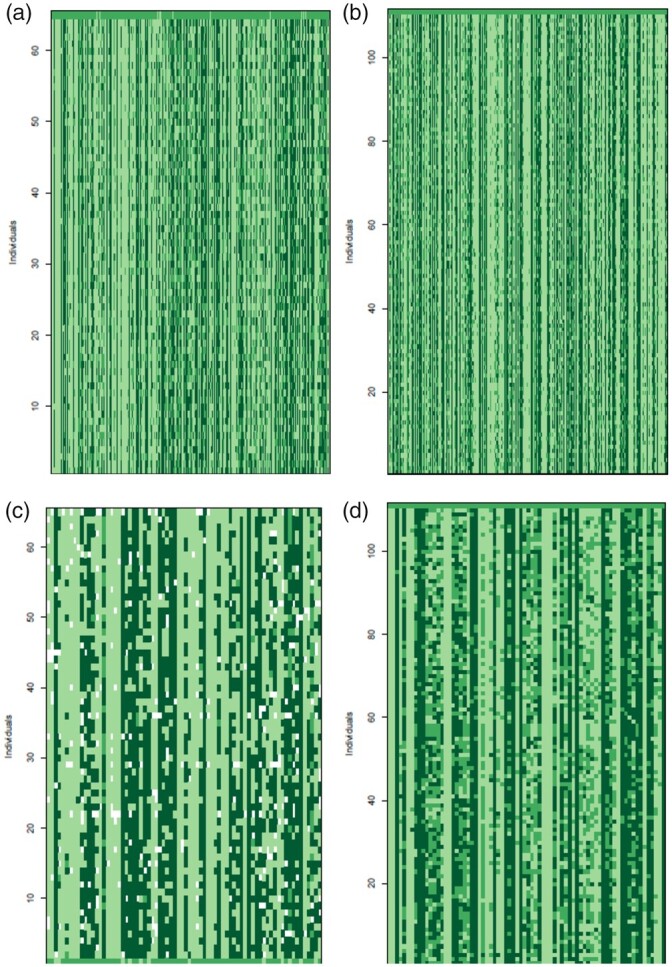
Allelic ancestry of markers that occur in hybrid populations for a) autosomal markers in females, b) autosomal markers in males, c) Z markers in females, and d) Z markers in males. Rows indicate individuals and columns indicate markers. Markers appear in chromosomal order. Dark green rectangles represent alleles that have an inland ancestry, light green rectangles represent alleles with a coastal ancestry, and medium green rectangles represent markers that are heterozygous for one allele of each type of ancestry.

Almost all markers tested had genomic clines that were significantly different from the neutral expectation of uninhibited gene flow across the hybrid zone. This suggests restricted gene flow between mitolineages, as is expected in the presence of RI (Table 3). Markers on the Z chromosome had significant cline values in males more often than in females; reduced power due to the hemizygosity of Z markers in females may have contributed to this pattern (Table 3). Patterns of allelic ancestry showed there to be extensive admixture within the hybrid zone for both males and females (Fig. 5). Individually significant genomic clines do not necessarily preclude admixture within the hybrid zone. Rather, nonassortative mating between mitolineages (i.e. if there is little or no prezygotic selection) will result in admixture throughout the nuclear genome in hybrid offspring, but selection against hybrid offspring (i.e. the presence of postzygotic selection) will cause genomic clines to show signatures of RI.

**Table 3.** Proportion of markers indicating RI.

	1A^Z^ markers	Autosomal markers	Z markers
Females	69/556 (12.4%)	885/902 (98.1%)	2/74 (2.7%)
Males	549/556 (98.7%)	902/902 (100%)	74/74 (100%)

Proportion of markers with genomic clines that differed significantly (*P* < 0.05) from the neutral expectation, indicating potential RI, between hybrid inland and hybrid coastal populations. The numerator indicates the number of loci with significant genomic clines, and the denominator show the number of loci tested in each category of marker and sex.

**Fig. 6.**
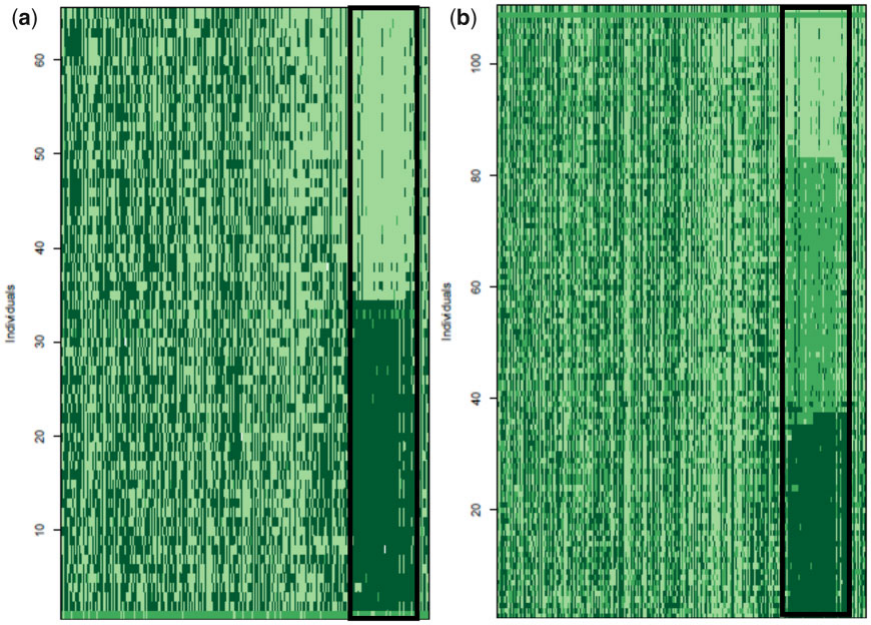
Allelic ancestry of 1A^Z^ markers for a) females that occur in the hybrid zone and b) males that occur in the hybrid zone. Rows indicate individuals and columns indicate markers. Markers appear in chromosomal order. Dark green rectangles represent alleles that have an inland ancestry, light green rectangles represent alleles with a coastal ancestry, and medium green rectangles represent markers that are heterozygous for one allele of each type of ancestry. The markers in the black outlined box represent genomic region 1A^Z^*.

Nearly all 1A^Z^ markers had significant clines in males, but in females this was true for only a minority (Table 3). As was the case with chromosome Z, this pattern is potentially a result of female hemizygosity. There was no admixture on a subset of these markers in females, while these same markers occurred in males either all as heterozygotes or all as homozygotes of either parental population (Fig. 6). This subset of markers thus appears to occur as a continuous block rather than independently segregating markers. In addition, the 1A^Z^ markers driving this population structure correspond to the 1A^Z^* markers that are most diverged between mitolineages, suggesting that the region of 1A^Z^* occurs in an area of low recombination. Whether or not an individual in the hybrid zone had an inland-type or coastal-type 1A^Z^* region did not strongly correlate with mtDNA. However, mismatching between mtDNA and 1A^Z^* haplotype was asymmetrical, with individuals bearing coastal-type mtDNA more likely to have an inland-type 1A^Z^* than individuals bearing inland-type mtDNA were to have a coastal-type 1A^Z^* (Table 4).

**Table 4.** Association between mtDNA and genomic region 1A^Z^* haplotypes.

		Inland 1A^Z^*	Heterozygous 1A^Z^*	Coastal 1A^Z^*
Males	Inland mitolineage	27/54 (50%)	23/54 (42.6%)	4/54 (7.4%)
	Coastal mitolineage	11/55 (20%)	21/55 (38.1%)	23/55 (41.8%)
Females	Inland mitolineage	16/24 (66.7%)	1/24 (4.2%)	7/24 (29.1%)
	Coastal mitolineage	18/40 (45%)	0/40 (0%)	22/40 (55%)

Proportion of inland-type, coastal-type, and heterozygous-type 1A^Z^* regions per mitolineage and sex in the hybrid zone. The numerator indicates the number of individuals with a particular combination of mitolineage and 1A^Z^*, while the denominator shows the number of individuals in a particular population.

While the shape of genomic clines relative to the neutral expectation can indicate if directional selection, epistasis, or over- or underdominance are acting on loci, these can be difficult to distinguish, and there was no singular, overarching pattern of cline indicative of any one type of selection (Gompert and Buerkle 2009). However, many clines did exhibit signatures of directional selection whereby clines are shifted left or right relative to that predicted by the neutral expectation (Supplementary Figs. 8–11, Gompert and Buerkle 2009). It was difficult to directly measure signatures of epistasis due to the expectation of very low heterozygosity in hybrids; however, this is unsurprising as epistasis only becomes visible in genomic cline analyses when selection is very strong and there are very large sample sizes in admixed populations (Gompert and Buerkle 2009). Still, large numbers of loci showed signs of probable underdominance, which is suggested when there is a sharper cline in homozygosity than that of the neutral expectation, although this can also occur with epistasis (Supplementary Figs. 8–11, Gompert and Buerkle 2009). A small number of loci exhibited signatures of overdominance, whereby there is a much higher probability of being heterozygous than predicted by neutral expectation, indicating that hybrids were under less selection than either parental genotype at these loci (Supplementary Figs. 8–11, Gompert and Buerkle 2009).

## Discussion

Resolution of biogeographic discordance in species that exhibit differing levels of divergence between their mitochondrial and nuclear genomes is an ongoing area of speciation research (Petit and Excoffier 2009; Toews and Brelsford 2012; Bonnet *et al.* 2017). The Eastern Yellow Robin, which is composed of 2 highly divergent mitochondrial lineages without concordant nuclear divergence, is one example of this, and the question of how putative nuclear gene flow between parapatric populations in this species is maintained without associated mitochondrial introgression has been questioned in other studies (Pavlova *et al.* 2013; Morales *et al.* 2017, 2018). Previous work has suggested that gene flow occurs between mitolineages, that incomplete lineage sorting does not explain the discordance between mitochondrial and nuclear divergence, and that speciation is driven by mitonuclear interactions between mtDNA and chromosome 1A (Pavlova *et al.* 2013; Morales *et al.* 2017, 2018). However, the results here suggest there is restricted gene flow between mitolineages (Table 3 and Supplementary Figs. 8–11). Impeded gene flow, in conjunction with the absence of evidence of assortative mating in field studies, indicates the presence of postzygotic but not prezygotic isolation between mitolineages (unpublished work). While Pavlova *et al.* (2013) found incomplete lineage sorting did not explain this discordance, the conclusions in the current study are based off of analyses involving approximately 10,000 times more markers than the previous work. Furthermore, the present study does not support the hypothesis that mitonuclear interactions are driving speciation. Rather, RI between mitolineages may be far too advanced to distinguish between markers that contribute to postzygotic isolation, as opposed to markers that have since diverged but did not themselves contribute to the initial process.

### Widespread signatures of impeded gene flow across the genome

Of the hundreds of loci tested, the vast majority exhibited clines that differed significantly from neutral expectation through the hybrid zone; this suggests selection against hybrids, as expected in the presence of RI, and indicates that the markers associated with RI are not restricted to particular areas of the genome (Table 3 and Supplementary Figs. 8–11). Because signatures of RI were so widespread across the genome, it is difficult to distinguish here which markers were contributors toward RI, and which have only begun to exhibit a signature of RI after speciation due to lack of gene flow (Nosil and Schluter 2011). The lack of gene flow associated with RI will cause marker divergence to increase; the low divergence currently observed across the genome is likely due to nonassortative mating and incomplete lineage sorting (Hudson and Coyne 2002; Cruickshank and Hahn 2014). While there does not appear to be any one type of selection driving RI between EYR lineages, the genomic cline analyses suggest selection against hybrids (potentially through genomic incompatibilities) and directional selection (putative local adaptation) may both be playing a role (Supplementary Figs. 8–11). In addition, some loci show genomic clines with signatures expected by overdominance (Supplementary Figs. 8–11). Overdominance is not necessarily incongruent with RI, as it is possible for F1 hybrids to exhibit heterosis, with the deleterious effects of hybridization occurring in the F2 and later generations (Edmands 1999; Gompert and Buerkle 2009). The widespread signatures of simultaneous RI and admixture, as well as nonassortative mating that has previously been observed in field studies, suggests that postzygotic but not prezygotic isolation is primarily driving speciation between the 2 EYR mitolineages.

### Haldane’s Rule a likely contributor of differentiation

There was a striking, sex-specific pattern with the markers that were differentiated within the hybrid zone between mitolineages, with many more markers exhibiting differentiation in females than in males (Table 2 and Fig. 4, a and b). Although a minority of the markers were differentiated in both sexes, most were differentiated only in one sex or the other (Table 2). Greater differentiation in female than male individuals in the hybrid zone is predicted by Haldane’s Rule, as epistatic interactions between the Z (and 1A^Z^) chromosomes and autosomes mean heterogametic females are more susceptible to the potentially deleterious effects of admixture than are homogametic males. Stronger selection against admixed females than males will result in females exhibiting stronger differentiation between mitolineages than males.

Stronger selection on females than males has previously been suggested to occur in EYR as a way to explain the strong divergence in mitochondrial but not nuclear genomes between inland and coastal populations (Pavlova *et al.* 2013). Mitolineages were suggested to be locally adapted to climate through mtDNA, and so selection on maternally inherited mtDNA and thus potentially its associated mitonuclear interactions would occur in females but not males (Pavlova *et al.* 2013; Morales *et al.* 2015). Indeed, theoretical and some experimental work predicts sex-specific selection is inevitable, as sexes should be adapted to independent optima, and maternal inheritance of mtDNA should drive sex-specific adaptation (Dowling *et al.* 2008; Connallon and Clark 2010; Connallon 2015). While the present study does not directly test for local adaption of mtDNA, it finds no evidence of sex-specific selection in the nuclear genome outside of that expected by Haldane’s Rule (Supplementary Fig. 5). However, if sex-specific selection is weak, and sample sizes are inadequate, it will be difficult to observe such phenomena, such as that in a wild avian system.

### Mitonuclear interactions on neo-sex chromosomes unlikely to be a key driver of divergence

Previous work found that EYR markers mapping to ZF chromosome 1A harbor a disproportionately high number of mitonuclear genes whose products interact and are in strong LD with those of mtDNA; these have been suggested to be coevolved so that individuals must have a copy of 1A that is functionally compatible with their mtDNA haplotype so as to avoid deleterious fitness effects (Morales *et al.* 2018). However, after consideration that chromosome 1A is a putative neo-sex chromosome, markers on 1A^W^ (which must necessarily be in LD with mtDNA) removed, and population structure taken into account, it is clear that 1A^Z*^ haplotypes can occur with the mtDNA haplotype of either mitolineage, both as heterozygous and homozygous genotypes (Table 4). While there is a trend for mitochondrial and 1A^Z*^ haplotypes to match, it is unknown if this is due to selection against admixture or some degree of assortative mating. However, the presence of individuals who have no 1A^Z*^ haplotype of the same mitolineage as their mtDNA indicates that there cannot be a lethal incompatibility between these 2 genomic regions. While most of the markers on 1A^Z^ were found to exhibit admixture, the markers within the genomic region of 1A^Z^* showed extremely high LD with each other, indicating it may be an inversion. While inversions are thought to allow coevolved alleles to be inherited together, such as those previously suggested to be involved in mitonuclear interactions, the region of 1A^Z^* has been previously found to contain few mitonuclear genes relative to ZF 1A as a whole (Ortiz-Barrientos *et al.* 2016; Morales *et al.* 2018; Wellenreuther and Bernatchez 2018, Morales H, personal communication). While it is possible that even one locus of large phenotypic effect could drive incompatibilities between 1A^Z^* and mtDNA, this should also cause strong divergence between mitolineages within hybrid populations and not allow for compatibility between 1A^Z^* and mitochondrial haplotypes; however, neither of these are seen here. Still, if loci on 1A^Z^ were indeed the driving factor in divergence between EYR mitolineages, with gene flow (and thus a lack of RI) occurring across the rest of the nuclear genome as has been previously suggested, markers on 1A^Z^ should exhibit the steep genomic clines indicative of RI, while markers on the rest of the genome would not (Morales *et al.* 2017, 2018). Rather, the great majority of loci tested exhibit genomic clines consistent with RI between mitolineages, as opposed to only those found on 1A^Z^. It thus appears unlikely that mitonuclear interactions between mtDNA and the derivatives of chromosome 1A (such as 1A^Z^) are strong drivers of speciation in the EYR.

Although mitonuclear interactions do not appear to be a strong driver of speciation in EYR, this does not necessarily discount the idea of mitonuclear interactions between mtDNA and the derivatives of chromosome 1A. It is possible that mitonuclear interactions between neo-sex chromosomes and the rest of the genome played an initial key role in driving divergence between mitolineages, but that the rest of the genome has progressed too far in postzygotic isolation for this mechanism to be observed in contemporary populations. Thus, while mitonuclear interactions may indeed be substantially involved in divergence, as is thought to have occurred in other avian species, other contributing biochemical pathways should not be ruled out; the large proportion of loci here showing signatures of RI make it difficult to discern what genes were involved in the original divergence, and there could be many factors at play (Trier *et al.* 2014; van der Heidjen *et al.* 2019). While the importance of mitonuclear coevolution in driving speciation, as well as evolution and ecology generally, has received significant recent attention, more empirical evidence is needed to determine how widespread this mechanism is across taxa (Hill 2015, 2019; Sloan *et al.* 2017).

### Different selective pressures operating in different geographic regions

Chromosomal rearrangements, including neo-sex chromosomes and inversions, have been implicated in speciation in a diverse array of taxa including birds (e.g. Kitano *et al.* 2009; Brooke *et al.* 2010; Pala *et al.* 2012; Bracewell *et al.* 2017; Hooper and Price, 2017; Hooper *et al.* 2019). It is possible a similar mechanism is operating in EYR, as the genomic region of 1A^Z^* exhibits an absence of markers with low *D*_XY_, which is commonly seen in inversions (Fig. 3, Cheng *et al.* 2012; Cruickshank and Hahn 2014). Neo-sex chromosome 1A^Z^ may be playing a role in divergence between EYR populations, as suggested by the high levels of *F*_ST_ and *D*_XY_ in 1A^Z^* relative to the rest of the genome (Fig. 3). However, because high levels of divergence are seen between allopatric but not parapatric populations, individual markers on 1A^Z^ may not be contributing to RI between mitolineages. Stronger differentiation outside than inside of the hybrid zone, as well as the lack of correlation between mtDNA and 1A^Z^* haplotypes, suggests that, although there may be little selective pressure for individuals to have coevolved 1A^Z^* and mtDNA haplotypes in the hybrid zone, there may be much stronger selective pressure for coevolved genomic regions in pure populations (Fig. 3 and Table 4).

While this study does not investigate the genes found on 1A^Z^* or its demographic history, inversions in particular can protect clusters of locally adapted alleles from recombination and are well established as playing a role in speciation (Berg *et al.* 2016, 2017; Ortiz-Barrientos *et al.* 2016; Barth *et al.* 2017; Wellenreuther and Bernatchez 2018). Strong local adaptation of 1A^Z^* is a potential explanation for why there is apparently little selection for an individual of a given mitolineage to have a coevolved 1A^Z^* haplotype: individuals within the narrow hybrid zone (and particularly at the same sites) must necessarily occur in similar climates, while individuals in pure populations do not. Additionally, the absence of a strong association between mtDNA and 1A^Z^*, as well as the presence of admixture throughout the rest of the genome in the hybrid zone, suggests very low recombination between the inland-type and coastal-type versions of 1A^Z^*. Low recombination between different 1A^Z^* haplotypes due to an inversion could presumably interfere with meiosis, thus rendering males who have heterozygous 1A^Z^* genotypes with reduced fitness. Difficulty with meiosis in the presence of interbreeding between mitolineages would also allow for all combinations of mtDNA and 1A^Z^* in the hybrid zone, while reduced fitness of heterozygotes would prevent introgression of 1A^Z^* haplotypes into opposing mitolineages. While further research is necessary to test if these hypotheses are true, they are capable of explaining the results of the current study.

### Future research directions

The results here show strong evidence of postzygotic isolation between mitolineages of EYR, and clear, sex-specific patterns of marker differentiation. Still, there remains much that is unknown. Detailed functional genomic studies will be needed to assess the biochemical pathways under selection so as to further understand the molecular basis of speciation and sex-specific selection in this system. Examination of phenotypic differences between mitolineages, as well as sexes, are required to confirm the traits under selection. Finally, the rate of hybridization between mitolineages and its fitness implications should be confirmed through intensive field studies, which are currently taking place, or through a controlled breeding program. While the EYR system is immensely complex, it stands to provide substantial advancements in speciation knowledge.

## Data availability

Supplementary Appendix 1 provides a list of individual identification numbers, mitolineage, sex, and sampling location of each sample. Supplementary Appendix 2 provides a summary of the number of DArT tags mapped to each ZF chromosome or linkage group. Appendix 3 contains data for the number of genotypes per sex and population per marker, as well as each marker’s mapped location to the ZF genome. Sequence data, as well as Supplementary Material Figures and all Appendices, have been uploaded using figshare doi: https://doi.org/10.25387/g3.19722793
